# Supplementary material for: Stable Production of a Recombinant Single-Chain Eel Follicle-Stimulating Hormone Analog in CHO DG44 Cells
Source: Int J Mol Sci. 2024 Jul 2;25(13):7282. doi: 10.3390/ijms25137282 (PMC11242883; doi:10.3390/ijms25137282)
Supplement: Supplementary file 1 [file ijms-25-07282-s001.zip › ijms-3027470-supplementary.pdf]

Stable Production of a Recombinant Single-Chain Eel Follicle-Stimulating Hormone Analog in CHO DG44 Cells

Munkhzaya Byambaragchaa, Sei Hyen Park, Sang-Gwon Kim,

Min Gyu Shin, Shin-Kwon Kim, Myung-Hum Park, Myung-Hwa Kang and Kwan-Sik Min*

*to whom correspondence should be addressed: ksmin@hknu.ac.kr

Supplementary Information

This .pdf includes:

Supplementary Figure S1

Supplementary Figure S2, S3, S4, and S5 (raw data of figure 2, 4, 5, and 9 in manuscript)

Supplementary Figure S1

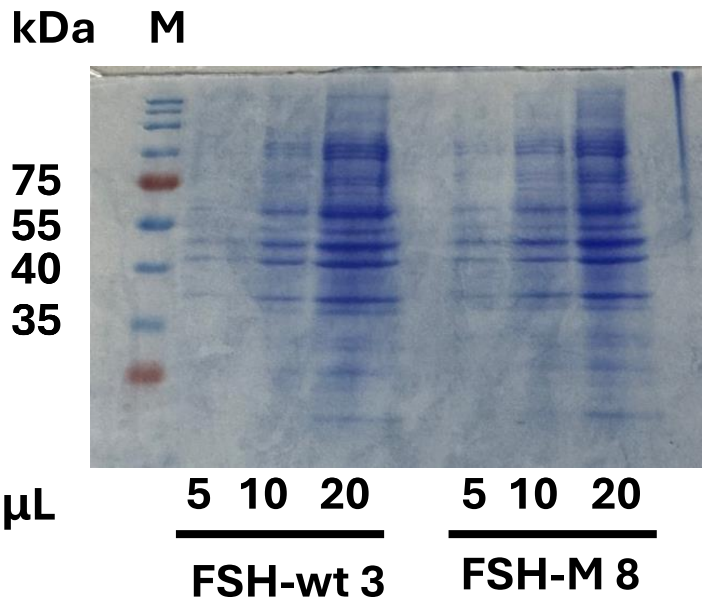

Supplementary Figure S1. Coomassie brilliant bule result of tethered rec-eel FSH-wt 3 and FSH-M 8 proteins. The supernatants of tethered rec-eel FSH-wt 3and FSH-M 8 were subjected to sodium dodecyl sulfate-polyacrylamide gel electrophoresis on a 12% reducing gel, following a Coomassie Brilliant Blue staining. The culture medium (5, 10, and 20 µL) of the tethered eel FSH-wt and FSH-M applied to the sodium dodecyl sulfate-polyacrylamide gel electrophoresis analysis, respectively. M: Marker; FSH-wt: wild-type of tethered rec-eel follicle-stimulating hormone; FSH-M: tethered rec-follicle stimulating hormone attached eCG β-subunit CTP linker.

Supplementary Figure S2 (figure 2 in manuscript)

(A)

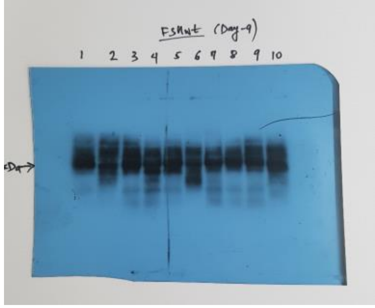

(B)

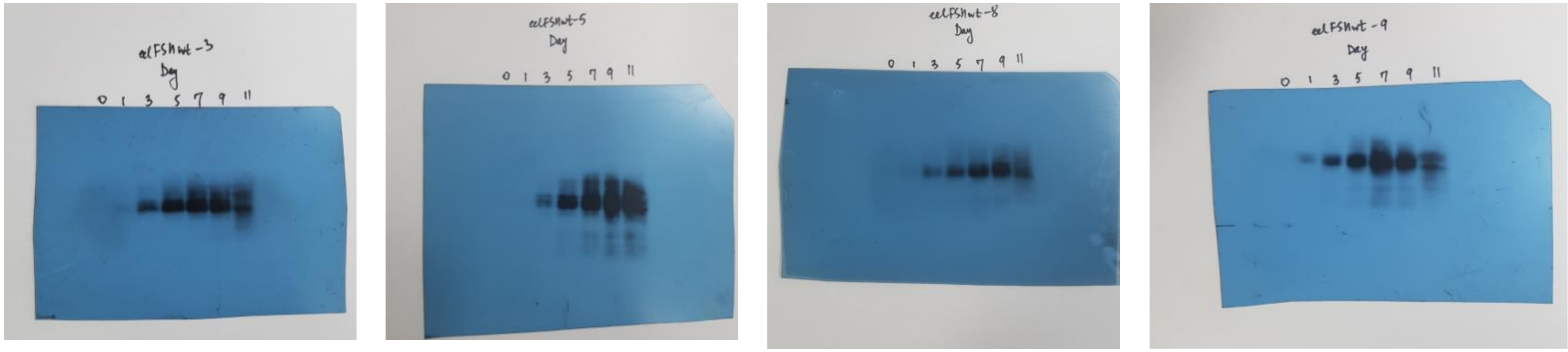

Supplementary Figure S2. Western blotting analysis of rec-eel FSH-wt proteins produced from single cells. (A) In total, 20 µL of the supernatant on day 9 was loaded in the wells. Numbers denote isolated clone counts. (B) Western blot analyses of four clones on the culture day. Four colonies (eel FSH-wt 3, FSH-wt 5, FSH-wt 8, and FSH-wt 9) were selected and 20 µL of supernatant was evaluated by western blotting on the day of culture. Proteins were detected using a monoclonal antibody (anti-eel FSH5A14) and horseradish peroxidase-conjugated goat anti-mouse IgG antibodies.

Supplementary Figure S3 (figure 4 in manuscript)

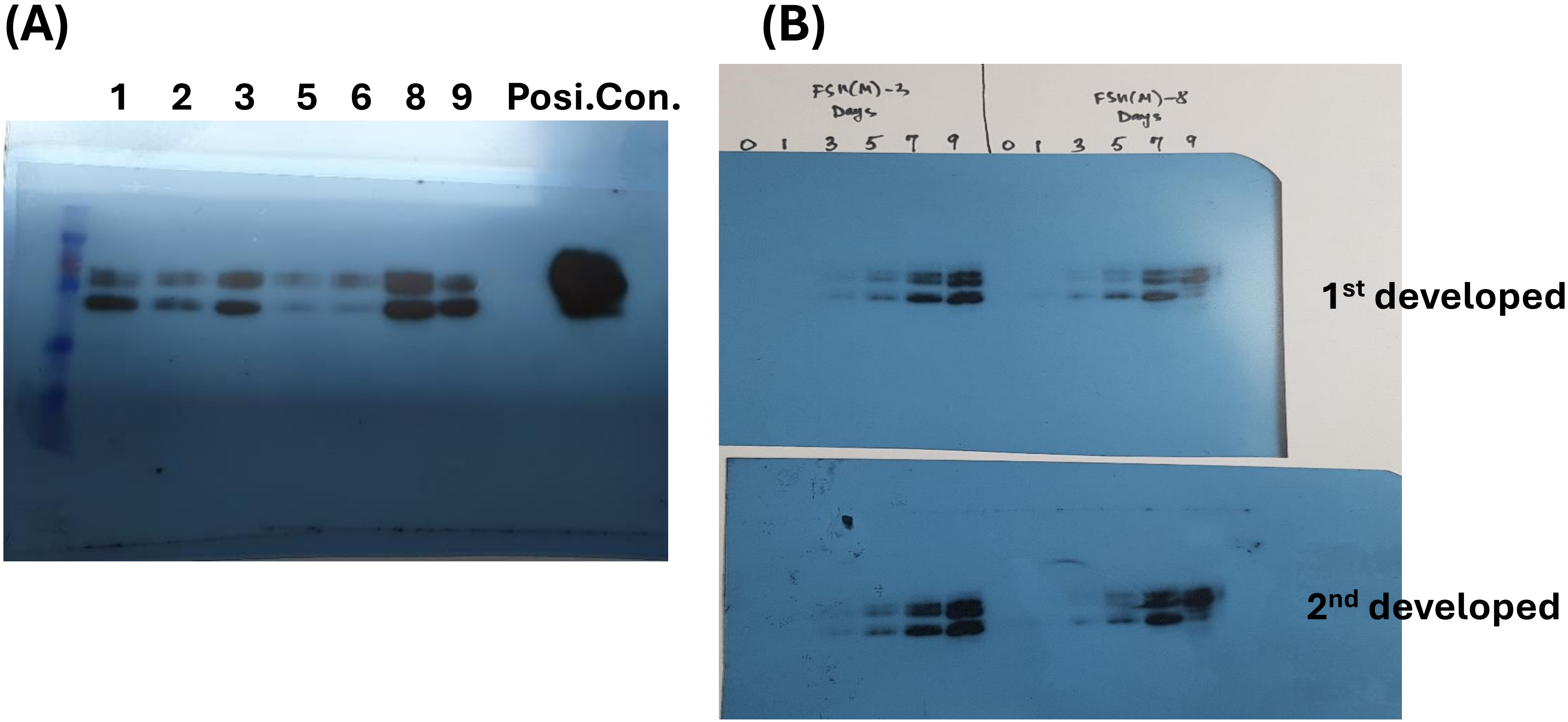

Supplementary Figure S3. Western blotting analysis of rec-eel FSH-M proteins produced by monoclonal cells. Supernatants were collected from seven colonies on the day of cultivation. After SDS-PAGE, membranes were detected using specific monoclonal antibodies (anti-eel FSH5A14). (A) In total, 20 µL collected on day 9 was loaded in the wells. Positive controls produced from the CHO-S cells were concentrated by 20 times and 20 µg was loaded in the wells. (B) Western blot analyses for FSH-M 3 and 8. Faint bands were first detected on day 3 and band intensity increased gradually, reaching a maximum on day 9.

Supplementary Figure S4 (figure 5 in manuscript)

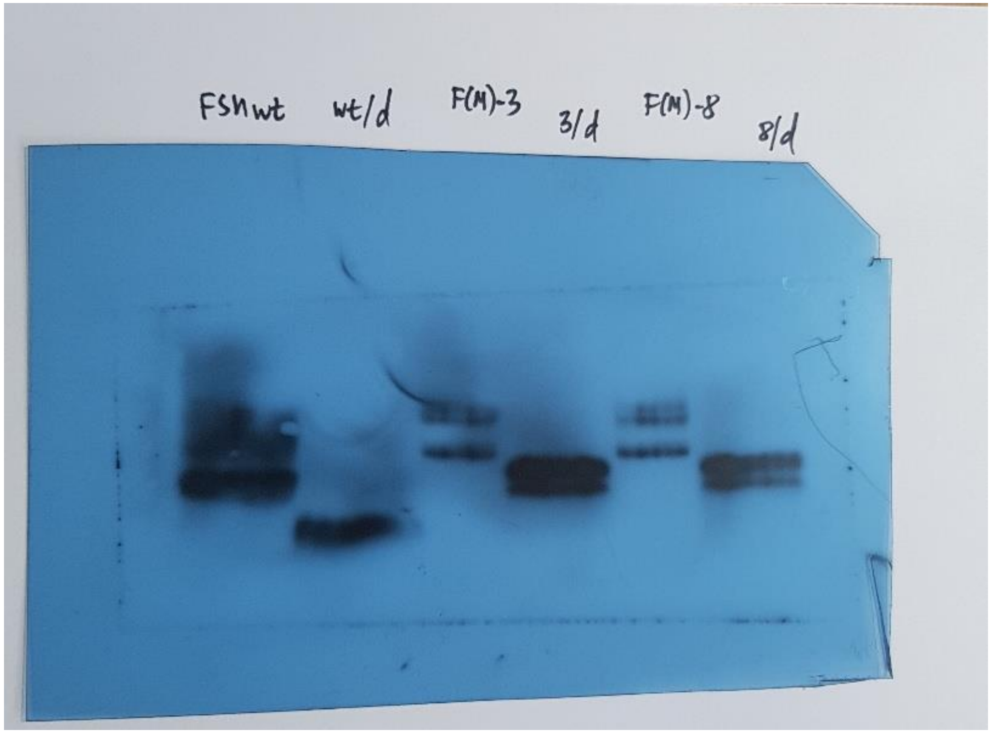

Supplementary Figure S4. Deglycosylation results for eel FSH-wt and FSH-M proteins. The proteins collected from tethered rec-eel FSH-wt 3, FSH-M 3, and FSH-M 8 were treated with peptide-N-glycanase F to remove *N*-linked oligosaccharides, followed by SDS-PAGE and western blotting by detection using the specific monoclonal antibodies (anti-eel FSH5A14). The molecular weights of tethered rec-eel FSH-wt and FSH-M decreased significantly to approximately 8–10 kDa.

Supplementary Figure S5 (figure 9 in manuscript)

(A) eel FSH-wt

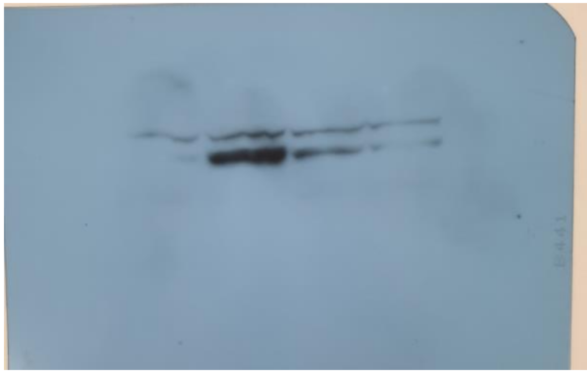

min 0 5 15 30

(B) eel FSH-M

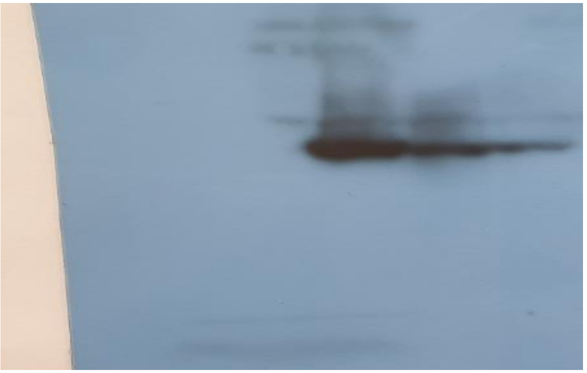

min 0 5 15 30

Supplementary Figure S5. pERK1/2 activation stimulated by eel FSH receptor. The eel FSH receptor was transiently transfected into HEK293 cells, and the cells were starved for 4-6 h and stimulated with a 400 ng/mL agonists for the indicated times. Whole-cell lysates were analyzed for pERK1/2 levels. Twenty micrograms of protein were used in each sample lane. The pERK and total ERK bands were quantified by densitometry, and pERK was normalized to total ERK levels. No significant differences were observed between the curves representing eel FSH-wt- and FSH-M-treated samples.
